# Brief admission (BA) for patients with emotional instability and self-harm: nurses’ perspectives - person-centred care in clinical practice

**DOI:** 10.1080/17482631.2019.1667133

**Published:** 2019-09-17

**Authors:** Joachim Eckerström, Emelie Allenius, Marjolein Helleman, Lena Flyckt, Kent-Inge Perseius, Pernilla Omerov

**Affiliations:** a Centre for Psychiatry Research, Department of Clinical Neuroscience, Karolinska Institutet, Stockholm, Sweden; b Department of Health Sciences, The Swedish Red Cross University College, Stockholm, Sweden; c Northern Stockholm psychiatry,Stockholm Health Care Services, Stockholm County Council, Stockholm, Sweden; d School of Nursing, Hanze University of Applied Sciences, Groningen, The Netherlands; e The Department of Health Care Sciences, Ersta Sköndal Bräcke University College, Stockholm, Sweden

**Keywords:** Borderline personality disorder, brief admission, crisis intervention, emotional instability, mental health nursing, patient admission, person-centred care, psychiatric nursing, self-harm

## Abstract

**Purpose**: Emotional instability and self-harm pose major problems for society and health care. There are effective interventions in outpatient care, but when patients need inpatient care, nurses often struggle meeting their patient’s needs. Brief admission (BA) is a newly implemented crisis intervention and novel form of inpatient care. The aim of this study is to describe nurses’ experiences working with BA related to patients with emotional instability and self-harm.

**Methods**: Eight nurses were interviewed according to a semi-structured interview guide. The data was analysed using qualitative content analysis.

**Results**: Four main categories emerged regarding nurses’ experiences with BA: *provides security and continuity, fosters caring relationships, shifts focus towards patient’s health* and *empowers the patient*. The nurse’s role shifted from “handling problems” to establishing caring relationships with a focus on the person’s health and possibilities for recovering instead of psychiatric symptoms.

**Conclusions**: Previous studies on patients’ perspective of BA describe positive experiences such as increased autonomy and participation in the healthcare process. This study supports those findings, albeit from the perspective of nurses. Our findings suggest that BA may reduce work-related stress experienced by nurses while caring for persons with emotional instability and self-harm. BA may also support nurses in their ability to provide more meaningful and constructive psychiatric inpatient care.

## Introduction

Emotional instability and self-harm cause major suffering for the affected persons and also pose problems for society and health care (Carroll, Metcalfe, & Gunnell, 2014). Although there are effective interventions in outpatient care (Links, Shah, & Eynan, 2017), nurses often struggle to meet their patients’ needs when patients require inpatient care (Westwood & Baker, 2010). Brief admission (BA) is a newly implemented crisis intervention and a novel form of inpatient care (Helleman, Goossens, van Achterberg, & Kaasenbrood, 2017). Previous studies have reported patients’ perspectives of BA for patients with emotional instability and self-harm (Helleman, Goossens, Kaasenbrood, & van Achterberg, 2014a, 2014b; Helleman, Lundh, Liljedahl, Daukantaite, & Westling, 2018). However, studies on nurses’ perspectives on using BA with different patient populations and in different settings are scarce. The aim of this study is to describe nurses’ experiences working with BA related to patients with emotional instability and self-harm.

## Background

### Self-harm

Self-harm is a major problem, especially among young people. A Swedish study involving more than 3,000 high school students indicated that 36% of them had hurt themselves at least once (Zetterqvist, Lundh, Dahlström, & Svedin, 2013). Self-harm is also a major problem for health care. A study conducted among people who had contact with mental health services reported that nearly half had harmed themselves in the last six months (Odelis & Ramklint, 2014). Self-harm is one of the most common reasons for emergency hospital admissions, and people presenting to hospital care after self-harm have an increased risk of suicide. Despite the magnitude of the problem, the incidence of repeated self-harm has not changed in over 10 years (Carroll et al., 2014).

### Emotional instability and borderline personality disorder

When self-harm is linked to emotional instability, which in its most pronounced form is diagnosed as borderline personality disorder (BPD), self-harm and suicidality often become part of a complex system of self-destructiveness that is difficult to manage, both for the individuals themselves and for healthcare professionals (Perseius, Ekdahl, Åsberg, & Samuelsson, 2005). BPD is characterized by a consistent pattern of instability in interpersonal relationships, self-image, and affect (American Psychiatric Association, 2013). The clinical signs of BPD vary, such as affective disturbance (rage, sorrow, shame, panic, terror, and chronic feelings of emptiness and loneliness), disturbed cognition (ideas of being bad), impulsivity (physically self-destructive, self-mutilation, suicidal communication, and suicide attempts), and intense unstable relationships (profound fear of abandonment, efforts to avoid being left alone, difficulties with close relationships, frequent arguments, repeated break-ups) (Lieb, Zanarini, Schmahl, Linehan, & Bohus, 2004). About 10% of patients diagnosed with BPD die from suicide (Black, Blum, Pfohl, & Hale, 2004; Paris, 2002). The health-related quality of life of those affected is reported to be extremely low (Perseius, Andersson, Åsberg, & Samuelsson, 2006), and feelings of hopelessness, self-loathing, and emotional distress are common (Perseius et al., 2005; Perseius, Öjehagen, Ekdahl, Åsberg, & Samuelsson, 2003). The emotional distress associated with BPD sometimes become unmanageable, and hospitalization due to self-harm and suicide attempts is common (Bolton, Pagura, Enns, Grant, & Sareen, 2010; Victor & Klonsky, 2014). However, inpatient care to address emotional instability and self-harm is often described as insufficient both by healthcare professionals and patients (Cleary, Siegfried, & Walter, 2002; Ejneborn Looi, Engström, & Sävenstedt, 2015; Holm, Björkdahl, & Björkenstam, 2011; Linehan, 1993; Perseius et al., 2003).

### Inpatient care

Inpatient care often results in increased stress and self-harm that severely affects patients and their health processes (Holm et al., 2011; Linehan, 1993). Patients frequently end up in long inpatient stays where coercive measures are common, removing autonomy and self-care as viable options (Perseius et al., 2005, 2003). Studies that focused on healthcare professionals’ experiences caring for persons with BPD found that nurses may have difficulty meeting their patients’ needs (Betan, Heim, Zittel Conklin, & Westen, 2005; Cleary et al., 2002; Westwood & Baker, 2010). Furthermore, nurses often perceive the patient-nurse relationship as problematic and sometimes “counter-therapeutic” instead of supportive (Dickens, Lamont, & Gray, 2016). Nurses working in inpatient care often meet these patients during their worst turmoil. Anger, self-harm, and suicide risk are challenging and often add to nurses’ emotional strain (Cutcliffe & Barker, 2002). Traditionally, inpatient psychiatric care often results in conflicts among patients as well as care personnel, which have negative effects on the ward environment and care (Newton-Howes & Mullen, 2011). Nurses are often involved in coercive measures that raise ethical concerns even when such measures appear necessary (Happell & Harrow, 2010). Limiting patients’ autonomy by restricting or denying requests has been shown to be the most important antecedent to violence and aggression within psychiatric inpatient care (Papadopoulos et al., 2012). Negative attitudes from psychiatric hospital staff towards inpatient care of patients with BPD may be partly attributed to the fact that staff are described as “the boundary keepers on the ward,” meaning that they have intense and restrictive interactions with patients in crisis (Bodner et al., 2015). Furthermore, nurses’ lack of control over their caseload may also lead to reduced empathy and more antagonistic attitudes. Systematic reviews of healthcare professionals’ attitudes towards caring for patients who self-harm show that negative attitudes are common (Karman, Kool, Poslawsky, & van Meijel, 2015; McHale & Felton, 2010; Saunders, Hawton, Fortune, & Farrell, 2012). However, a more positive attitude was associated with an increased understanding of self-harm and improved training (McHale & Felton, 2010); with active training and working in mental health care as compared with general care; and with higher education, working in mental health care, and working in outpatient care (Karman et al., 2015).

Previous studies show that working with a structured method such as dialectical behavioural therapy may reduce work-related stress among healthcare staff (Perseius, Kåver, Ekdahl, Åsberg, & Samuelsson, 2007). In recent years, psychological treatments have been developed that can increase a patient’s ability to handle symptoms of emotional instability (Links et al., 2017). However, these interventions were developed for outpatient care instead of inpatient care. The focus on the psychological and pharmacological treatment of symptoms rather than on mental health nursing has also been suggested as one explanation for deficient patient-nurse relationships, which are characteristic of psychiatric inpatient care (Cutcliffe, Santos, Kozel, Taylor, & Lees, 2015; Jonsson et al., 2014).

### Patient-nurse relationship

A review on nurses’ attitudes towards self-harm found that training and education as well as support and time are needed to create the therapeutic relationship necessary for providing high-quality care (Karman et al., 2015). Langley and Klopper (2005) explored the therapeutic relationship between patients diagnosed with BPD and psychiatric nurses. Both patients and clinicians identified trust as a foundation for a therapeutic relationship. To develop a trustful patient-nurse relationship, the following characteristics were highlighted as important for nurses: appears available and accessible, listens and tries to understand, seems caring, creates an emotionally and physically safe environment for the patient, fosters professional and honest interactions, respects confidentiality, treats the patient as an adult, and acts calm (Langley & Klopper, 2005). Another extensive review of the attitudes, behaviours, and experiences of mental health nurses who work with patients with personality disorders concluded that new innovative approaches are needed to improve patient-nurse relationships (Dickens et al., 2016).

### Brief admission (BA)

Brief admission (BA) is a newly implemented nursing intervention for psychiatric inpatient care that may be used for patients with emotional instability and self-harm, including those diagnosed with BPD (Helleman et al., 2014a, 2014b). Studies of BA with other patient groups report that patients who suffer from psychosis (Heskestad & Tytlandsvik, 2008; Rise et al., 2014) and anorexia (Strand, Bulik, von Hausswolff-Juhlin, & Gustafsson, 2017) showed positive experiences such as increased autonomy and participation in the healthcare process as well as an increased sense of control and security in handling crises in a constructive way. The relationship with nurses is often described as essential in patient evaluations of BA (Helleman et al., 2014a, 2014b).

The intervention used in this study was developed from the Dutch model for BA (Helleman et al., 2014a, 2014b). According to this model, creating the prerequisites for helping patients manage their symptoms as well as achieving increased autonomy and self-esteem are key elements of the intervention (Helleman et al., 2014a). Furthermore, *the purpose of BA* is “to provide a time-out for self-management in a safe environment in situations of increased stress and threatening crisis,” and *the aim of BA* is “to promote the patient’s constructive coping strategies and thereby prevent self-destructive behaviour as well as prolonged admissions” (Helleman et al., 2014a). According to the model, BA is initiated by the patient, lasts for no more than three days for any single admission, and is available up to three times per month. The BA approach is managed entirely by nurses from admission to discharge.

### Care structure of BA

BA focuses on supporting patients towards finding a balance between daily activities and relaxation, and patient rooms must be allocated for BA in advance for when patients need them according to the BA contract (intervention care plan) (Helleman et al., 2014a, 2014b). The care environment is important to set “the tone of care” for both the patients and healthcare personnel (Karlin & Zeiss, 2006). The importance of creating a safe yet attractive environment that secures privacy has been demonstrated in the literature (Browne, 2013).

**Figure 1.**
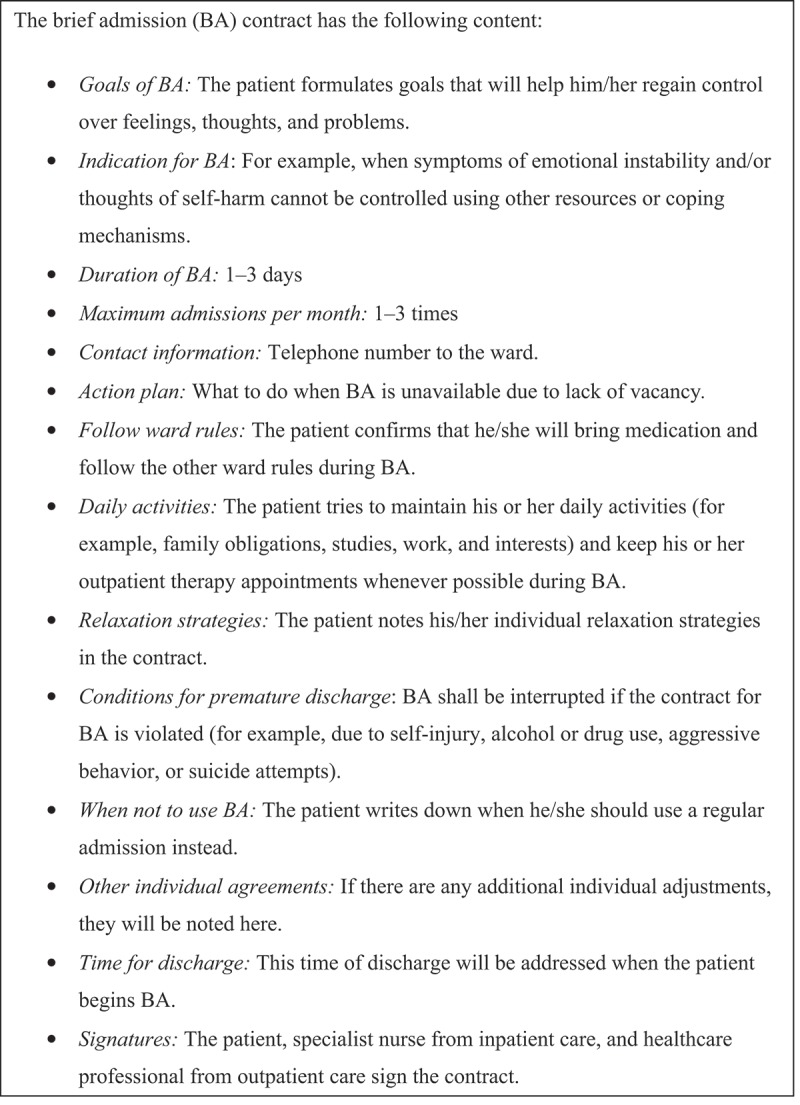
Content of the brief admission (BA) contract.

The first step of BA is the contract (Figure 1) negotiation process, in which a written contract is drafted outlining the BA care process after a thorough discussion between the healthcare staff and the patient. This step is an important aspect of BA because the contract provisions set forth the patient’s care plan and crisis plan (Helleman et al., 2017). A central feature of this step is that it is completed and signed by the patient, a specialist nurse from inpatient care, and a healthcare professional from outpatient care, with all parties agreeing on the specific details relating to the care support to be provided. The contract drafting process is, like every aspect of BA, characterized by mutual respect and cooperation (Helleman et al., 2014a, 2014b).

## Methods

This study adopted a qualitative approach to capture the nurses’ subjective experiences with BA for patients with emotional instability and self-harm. The data were collected through individual interviews and analysed using qualitative content analysis (Elo & Kyngäs, 2008).

### Setting and participants

BA was implemented in January 2016 in a psychiatric clinic in Stockholm in connection with a larger ongoing research project. This qualitative study focuses on the nurses’ perspectives on BA in a ward specializing in patients with emotional instability and self-harm that had a single room customized for BA. Patients eligible for BA displayed clinical signs of both emotional instability and self-harm, although not necessarily fulfiling all criteria for a BPD diagnosis. At the time Purposive sampling was used to obtain nurse informants who could provide relevant data for the study’s aims and objectives. Inclusion criteria for the nurse informants were those who work day shifts as registered nurses on the ward with BA. Ten nurses fulfiled the inclusion criteria and were invited to participate by email, in which an information letter about the study was included. Eight of them chose to participate, seven women and one man. The participants’ work experiences as registered nurses varied between one to 33 years. Four of the participants were either specialist nurses in mental healthcare with a master of science degree in nursing or currently working on their specialist degree.

### Professional approach during BA

Before implementation of BA in the ward, all of the ward’s healthcare professionals were educated on the intervention’s contents. The professional approach that is emphasized during BA is characterized by warmth, acceptance, genuineness, openness, confirmation of current difficulties experienced by the patient, and willingness to cooperate on equal terms with the patient. The patient can define by contract the frequency of contact he or she wants with the nurse and other healthcare professionals during BA. At admission, the nurse freely welcomes the patient to the ward; for example, there is no searching of the patient’s clothes or bags. Conversation and contact are offered according to the terms of the contract. If the patient wishes to change medications or their ongoing contacts with outpatient care, the nurse will assist them in planning how to contact the outpatient facility after discharge. At patient discharge, the nurse discusses the present admission with the patient, and together, they plan how they can increase the patient’s autonomy in future admissions, based on current knowledge.

### Data collection

The research team created a semi-structured interview guide. One pilot interview was used to assess whether the questions were appropriate and comprehensible, leading to minor revisions. After written informed consent was obtained from the informants, the interviews began with background questions about education and work experience. The informants were then encouraged to freely discuss their experience of BA. The interview guide consisted of questions covering themes related to the nurses’ experiences working with BA and how it affected them as well as their work with patients and other team members. The interviews were conducted by the second author (EA), an experienced nurse educated in qualitative research methods. The time and place for the interviews were selected by the informants. Seven of the interviews took place at the psychiatric hospital, and one was carried out in a public place chosen by the informant. The interviews took place from December 2016 to March 2017 and lasted between 40 and 74 minutes; they were audio recorded digitally and later transcribed verbatim (MacLean, Meyer, & Estable, 2004). At the time of the interviews, 27 patients had the opportunity to use BA on that ward.

### Data analysis

To analyse the data, the research team used qualitative content analysis with an inductive approach as explained by Elo and Kyngäs (2008). The analysis involved open coding of the transcripts and an iterative generation of sub-categories, generic categories, and main categories through discussion among the researchers. The analysis ended with a summarization, which is presented in the findings. The accuracy of the transcriptions was confirmed by comparing the recordings with the transcriptions. The authors (JE, EA, KIP, and PO) read the transcriptions to obtain an understanding of each transcript and the content. Content that was relevant to the aim of the study was marked, labelled, and described in the open-coding process. New categories and sub-categories were formed by comparing the similarities and differences in the information identified during open-coding. The information retrieved from the eight interviews was assessed as sufficient to fulfil the aim of the study. The abstraction process resulted in two main categories. The authors worked independently with the analysis, and the emergent results were continuously discussed, refined, and agreed upon within the author group.

### Ethical considerations

The study followed the ethical standards of the World Medical Association’s Declaration of Helsinki (2013) and was approved by the Regional Ethics Committee of Stockholm, Sweden (2016/671-31/5). Following the regulations and guidelines of Sweden and the EU standards for research involving humans, the participants were informed about the study, that participation was voluntary, about the handling of personal data, that personal information would be kept confidential, and that the results would be presented as a master’s thesis as well as in scientific papers. Written informed consent was obtained prior to the interviews. To protect the participants’ confidentiality, personal information that could be used to identify the participants was omitted from the interview material. The risk of harm related to research participation was considered minimal while the opportunity to share experiences was considered beneficial, both for the participants and the field.

## Findings

Qualitative content analysis of the interviews with the nurses who had worked with BA revealed four main categories: (1) provides security and continuity, (2) fosters caring relationships, (3) shifts focus towards patient’s health and (4) empowers the patient. Each main category had generic categories, followed by sub-categories and codes (Table I).

**Table I.** Categories from qualitative analysis of nurses’ experiences with brief admission (BA).

Main categories	Provides security and continuity	Fosters caring relationships	Shifts focus towards patient’s health	Empowers the patient
**Generic categories** *– Sub-categories*	**Knowing what to expect** *– Fewer disappointments for patients and nurses* *– Patients have more control* *– Structure enables care planning*	**Support, not “handling problems”** *– Nursing instead of mediating* *– Nursing instead of medicating* *– Patient-nurse conversations take priority*	**Personalized structured care** *– Person in focus, not diagnosis* *– Person in focus, not treatment* *– Focus on health and possibilities*	**Towards** **partnership** *– Partnership instead of hierarchy* *– Patient as a co-worker on the team* *– Challenging and demanding to make the shift*
	**Limits regulated by contract, not nurse** *– Fewer patient-nurse conflicts* *– Reliance instead of control*	**Towards** **caring relationships** *– More trustful and equal relationships* *– Peace to work forward* *– Increased and shared understanding* *– Painful when the relationship became too close*		**Towards** **equal value** *– Mutual respect for each competence* – *Acknowledging the patient’s knowledge*
	**Fewer patients improved relationships** *– Fewer patients led to fewer misconceptions* *– Continuity enables forming relationships*			

### Provides security and continuity

Based on the BA approach, only a few people were involved in patient care from admission throughout the inpatient stay. Instead of passing through the somatic or psychiatric emergency department chain of care, the assigned nurse met the patient directly, and the care started during the initial meeting. The nurses reported that this new approach to admission had several positive effects on the patient-nurse relationship. The direct communication between the assigned nurse and patient reduced misunderstandings that are sometimes generated when communications are filtered through several nurses and physicians.

“It is me who has taken this conversation and I receive [it] and then it is up to me to get it to work all the way. There are no intermediaries, and this probably leads to a much better treatment, and you will have a more direct communication.” (Participant number 5)

Knowing that the patient will be admitted for an agreed upon length of time provided a positive starting point, which is different from the uncertainty associated with admissions that result after numerous encounters with different staff. In addition, the nature of the admission process for BA differed from the regular admission process. The nurses provided examples of previous experiences during which admissions were based on self-harm and threats of suicide. The nurses sometimes felt that the patients exaggerated their current symptoms to secure inpatient care that the patient felt was necessary. The nurses felt that the patients appreciated the ability to be admitted in BA when they perceived that they needed inpatient care instead of their needs being scrutinized and determined by a physician. The nurses reported how BA changed the admission process from a time-consuming struggle to a prompt meeting that was “looked after” more carefully and “taken more seriously.”

The predetermined focus of care and length of inpatient stay reduced arguments and conflicts that commonly arise during regular admission. The nurses provided examples from earlier experiences of conflicts with patients who were disappointed with their pharmacological treatment or patients who tried to influence the time of discharge. During BA, the patients were responsible for administering their prescribed medicines. This meant that the nurse was not the one who decided if the patient would receive his or her prescribed medications. Furthermore, according to the BA contract, all discussions about changes in treatment were to be held in the patients’ regular care. The absence of pharmacological discussions meant avoiding anger and frustration, which are common when patients’ expectations cannot be met by a nurse. Additionally, the nurses described their new responsibility as a challenge because they had to support patients with nursing interventions instead of medications.

“As a nurse, you must step up a bit, because it is much easier to give someone an Oxascand [a short- to intermediate-acting benzodiazepine] than to sit and talk, although it is more rewarding to talk … .” (Participant number 4)

The nurses also reported how they sometimes found it difficult to trust the patients’ self-management instead of having a more controlling role, to which they were accustomed. The mutually established contract helped the nurses change their approach, allowing them the possibility of referring to the contract provisions when there were disagreements between the nurse and patient. This led to fewer unfruitful discussions about medications and other courses of treatment, which the nurses believed led to more constructive behaviour during the inpatient stay. The contract also enabled the patients and nurses to create nursing plans that could be successfully implemented. The nurses contrasted this with previous experiences with care plans that were abruptly terminated when the patient was considered medically finished and discharged by the physician.

“Otherwise you do not always know, the patients may be discharged before you …, yes before or after or the plan changes or something. It is more unpredictable outside brief admission and as a result you must be very flexible the whole time. While here you can be pretty like this, you can count on this predictability, you can promise predictability and continuity, and you can live up to it. Which you cannot always do during ordinary admission.” (Participant number 6)

### Fosters caring relationships

The nurses described how BA shifts the focus from medication and “handling problems” to forming a caring relationship with the patient. The improved patient-nurse relationship leads to increased understanding about the person behind the patient, which was mostly described in a positive way. However, one nurse stated that the deepened relationship was painful because he/she became too close with the patients. BA gave the nurses the mandate and possibility of focusing on nursing care. The nurses reported how their work changed towards supporting patients instead of playing the role of mediator between the physicians and patients. The nurses described how their opportunity to communicate with patients improved with BA. In regular admissions, their conversations with patients are often limited to unplanned conversations that are interrupted by other duties, which resulted in mostly quick conversations in the corridors. The nurses welcomed the structure of BA because patient-nurse relationships and communication became an important focus within the organization. The scheduled and carefully planned meetings resulted in more respectful and quality meetings, according to the nurses. The focus of the conversation shifted towards what the patient wanted to talk about, and staff continuity lead to deeper conversations and a better understanding of the patient. This was accomplished even though admissions were shorter for BA than other forms of admission.

“Since there are several [patients] who come in repeatedly, I get a pretty good idea of what is stressful in their lives, what hopes they have, what they are sad about, and what is specific about this admission. So, it’s somehow a little deeper contact in that way, even if it may only be a couple of days.” (Participant number 8)

“The patient is more receptive to the information, so it becomes another sort of relationship really, it becomes a professional relationship of course, but nevertheless you can follow-up in another way because these patients are often returning.” (Participant number 1)

The nurses noted the difference in working with patients who were motivated for the stipulated care. Disappointments such as having to wait for BA when the rooms were full or unfulfilled promises during BA were often met in a constructive way by the enrolled patients. The nurses spoke about changed roles, both for themselves and the patients. The nurses’ role was described as providing a “helping hand.” They also described how their view on receiving recurring patients changed from negative to positive and how they genuinely encouraged their patients to seek help and emphasized that they were not causing trouble.

“You have somehow considered that this is something chronic that cannot be helped: ‘This is how it will be,’ ‘these patients are like this,’ ‘it’s hopeless,’ ‘it’s getting better over the years,’ sort of. But now it shows, so clearly, that it’s possible to do something about and improve … yes, you must really give this patient group a chance. Not to feel like ‘this is condemned to fail’ but instead raise these patients and show them ‘it is possible,’ ‘you can,’ and strengthen them.” (Participant number 1)

The increased understanding and deeper relationships also resulted in respect for the other person’s experiences and competences. The nurses described situations in which the patients took responsibility for their moods and actions, resulting in a positive response from the nurses.

“You can see when they are doing well and take control and feel good. You can see that part and can sort of support the positive part, instead of one being just like a border or ending up in between and being the one who poses the limits. So, I absolutely believe that you have gained a more positive relationship with patients, some patients, after that.” (Participant number 6)

### Shifts focus towards patient’s health

The patient’s personal needs and possibilities became the focus instead of “the emotional personality disorder or risk of self-harm.” The patients’ psychiatric needs were superseded by their personal care needs during the BA intervention, and the nurses described how this resulted in a shift of power towards a more equal relationship between nurses and patients.

“It has been very interesting to see how the same patients respond to being ‘BA patients’ instead of ‘ordinary patients’ … it becomes a change, especially for some, which is very positive. They feel a little more strengthened, more responsible, take a little more initiative. And I think that you already notice that the first time they use Brief Admission.” (Participant number 6)

Several of the informants reported that the patients’ self-awareness increased when the daily conversations target health promoting actions, such as how the patient can constructively improve their mental well-being. Because there is a clear treatment plan in the BA contract, the treatment did not become the topic of discussion; instead, the healthcare professionals supported the patients in using their own resources, re-establishing routines, and seizing opportunities to make every day matter. One informant highlighted that the patients took their responsibility and that previous preunderstanding about the usage of BA wasn’t answered.

“I had from the beginning doubts that it [BA] would be used in the wrong way and just this with daily conversations and that they would come and ask for conversations constantly and that they would get longer and longer and stuff like that and that they would refuse to go home. So, it hasn’t been. Instead it been very, ‘have we decided that discharge will be eleven o’clock three days earlier, then it will be at eleven o’clock three days later’. And it has worked very well, I think” (Participant number 7)

The informants attributed this shift towards the patients’ health as improving the patients’ motivation, will and initiative in their care. The positive and encouraging attitude towards the patients as well as an optimistic view of the patients’ ability to recover were considered important factors for the patients’ future health status.

### Empowers the patient

The shift in focus and the transparency and openness in the care planning elevated the patients into the group of the “most knowing,” a role usually reserved for physicians. The nurses noted that the hierarchy often found within psychiatric inpatient care was replaced by a feeling of partnership, where the patient became a respected co-worker. The nurses noticed that the absence of a physician led to a greater focus on nursing and increased responsibility for the nurses and patients.

Although this increased level of responsibility was welcomed by the nurses, it was accompanied with uncertainty over decisions that they were now required to make. They had fears of self-harm and suicide, which made decision making “challenging, demanding, and worrying.” The importance of a clear framework for inpatient care during BA was emphasized. During regular psychiatric inpatient care, the nurses often observed and controlled patients according to recommendations for suicide prevention. The nurses found it challenging to balance patient trust with observation, for example, during times when the patients’ moods were altered.

“Since you are used to the regular inpatient care patients, you are used to keeping an eye on everyone and that you do not have in the same way [during brief admission]. Then … I do not know … . A need of control, that you should let them go a lot more and it’s good that you should do that, but it can be difficult.” (Participant number 5)

“It is their own responsibility, this is brief admission ‘yes, but think if she runs away and jumps or … ’ then it will be my responsibility during the night. So, there are a lot of [thoughts] like that that are circulating, that you are uncertain.” (Participant number 1)

The transfer of responsibility to the patient and the relinquishing of control were, nevertheless, described as keys to success. The nurses reported that trust in the patients’ capacity led to more constructive and responsible behaviour because the patients took more initiative for their care.

“To take responsibility for their own well-being, their recovery, means that you get better confidence in that particular management. You get strong in that regard, ‘yes, I can actually manage to take my medication and be admitted on the ward without such as forced injections and people who should run and do everything for me all the time’”. (Participant number 3)

The nurses emphasized a more positive, warm, and welcoming atmosphere towards patients with emotional instability and who self-harm and suggested that BA played an important part in this much-needed development.

By making the patient a co-worker, BA promotes mutual respect for each other’s competence. Nurses observed more mature and adult behaviour in patients during BA compared to patients during general admission. The patients do not need to persuade or convince staff or exaggerate symptoms to be admitted; instead, they make the decision themselves and ask if BA is available. The nurses explained that with a general admission, there is likely more drama around the patient, such as self-injury and suicidal threats expressed by the patient. By contrast, the nurses described that with BA, hospitalization is a positive process because the patients have already acted in a constructive manner by seeking admission. Some of the nurses also perceived that BA patients are able to handle negative responses in a positive way, such as when a patient is denied admission because the BA room is already occupied or is denied additional support calls over what was provided for in the contract.

## Discussion

To our knowledge, this is the first study to focus on psychiatric nurses’ experiences with BA for patients with emotional instability and self-harm. The nurses in the study described how BA improved the patient-nurse relationship by providing a structure for security and continuity throughout the inpatient stay. Our findings also show that BA mandated that nurses focus on forming a caring relationship with the patients instead of just “handling problems.” The nurses described how BA established a positive context that affected the nurses and also the patients, such that they worked together constructively as a team. The reported outcomes of the improved patient-nurse relationship were described in terms that define person-centred care, suggesting that BA may be an intervention that promotes person-centred care.

### Improved patient-nurse relationships

The BA contract prepared the nurses and patients for what to expect during the inpatient stay, resulting in “fewer disappointments” according to the nurses. The nurses welcomed that the limits were regulated by contract and not themselves personally. Fewer people involved in the care also led to fewer misunderstandings. The nurses acknowledged the difference in caring for patients who were not in acute psychiatric distress or vulnerable to self-harm. Our findings resemble those of a recent Danish multi-centre study on patient-controlled psychiatric hospital admissions (Ellegaard, Bliksted, Lomborg, & Mehlsen, 2017a; Ellegaard, Mehlsen, Lomborg, & Bliksted, 2017b). The Danish study showed that both patients (n = 190, 462 admissions) and mental health professionals (n = 252, 546 questionnaires) rated the intervention of short patient-controlled admissions (PCA) as positive. The patients welcomed the agreement that enabled them to receive early assistance and avoid becoming too ill, thereby avoiding emergency admission. The study found that patients who had access to a shorter period of PCA were more satisfied than those who had access to a longer period of PCA. Furthermore, a quarantine period, which was a required period of time before a new PCA period could begin, was associated with better preparedness for discharge compared with no quarantine period. Nevertheless, one third of the patients reported that they did not feel ready for discharge when they were required to leave the ward (Ellegaard et al., 2017a). Contrary to those findings, the nurses in our study did not describe the limitations on inpatients days or available beds as problematic. Neither did the nurses in our study describe any issues based on the BA contract not being drafted with the patient as an equal care partner. Earlier studies on BA have suggested that guidelines may need to be established for the BA contracts (Strand & von Hausswolff-Juhlin, 2015). Our findings as well previous studies show that more research is needed to examine the advantages and disadvantages of the BA contracts. Future studies should examine the effects of BA contracts as well as experiences from both the patients and nurses’ perspective.

The nurses in the present study described that the BA concept changed the nurses as well as the patient’s roles towards constructive solutions and mutual respect. Consistent with earlier studies, arguments and conflicts between the nurses and patients were reduced through BA (Newton-Howes & Mullen, 2011), as well as negative attitudes (Bodner et al., 2015). The nurses described how they became more positive towards their patients after working with BA. The nurses also perceived that BA reduced self-harm, which may also result in an increased willingness to care for these patients. Nevertheless, the nurses still worried about the risk of self-harm and suicide during the inpatient stay. Some nurses found it difficult not to apply the regular measures for preventing the patients from harming themselves, and the nurses’ increased responsibility was described as stressful. At the same time, transferring responsibility to the patient and relinquishing control were described as critical to the success of BA. A nurse’s views on health and the patient affect how that nurse views his or her role. Barker and Buchanan-Barker (2010) emphasize that healthcare professionals must acknowledge that the patient is the captain of his or her life’s journey, even during times of crises when support from others is needed. Viewing the patient as a capable actor increases the chances that patients will also view themselves in this light. Suicide may be viewed as the result of a process where events lead up to an emotional crisis, upon which the person acts. In this way, suicidality is something that the affected person needs to work with and professionals may provide valuable support (Gysin-Maillart, Schwab, Soravia, Megert, & Michel, 2016; Michel, Valach, & Gysin-Maillart, 2017). According to the caring science perspective, the nurse must believe in the patient’s ability to evolve (Barker & Buchanan-Barker, 2010), which was fostered in our study.

The need for nurses to shift from psychiatric care towards mental health care is stressed by scholars (Cutcliffe et al., 2015; Jonsson et al., 2014). Focusing on physically preventing self-harm and suicide by “constant observation” may, for example, increase the patients’ emotional distress as well as hinder a caring relationship (Cutcliffe & Barker, 2002; Cutcliffe et al., 2015). It has been shown that using a structured method like dialectical behavioural therapy may reduce work-related stress in healthcare staff when working with self-harming and suicidal patients (Perseius et al., 2007). The findings in the present study indicate that BA might produce similar effects; however, this needs to be further investigated.

Our findings are in line with previous studies suggesting that BA enables psychiatric nurses to support patients in finding their own resources, which include resources that may prevent self-harm in patients with BPD (Helleman et al., 2014a). The nurses in this study described how BA created a welcoming atmosphere in which the nurses could focus on encouragement, which is similar to what Barker and Buchanan-Barker (2018) describe as supporting patients to “get going again”. The nurses noted that BA gave them a mandate and the possibility of working with their patients through conversations, which Karman et al. (2015) have suggested to reduce nurses’ negative attitudes when working with patients who self-harm. The nurses’ role changed from mediating between the psychiatrist and the patient to having quality conversations regarding the patient’s needs. Previous research showed that emotional instability and self-harm often create difficulties in a patient’s daily life (Helleman et al., 2017) and that patients may need short psychiatric hospital admissions when they have problems related not only to mental health conditions, but also to social and everyday problems. Nevertheless, patients who had hoped for changes to their pharmacological treatment were less satisfied (Ellegaard et al., 2017a). In our application of BA, nurses supported patients who wished to change their medications during BA by establishing contact with their outpatient care providers after discharge, which the nurses found effective. We do not, however, know the patients’ experience with this aspect of BA due to our study design.

### Promotes person-centred care

The nurses reported that BA shifted the focus from psychiatric aspects towards a focus on the patients’ health and possibilities. BA required that the nurses release control to the patients, and the nurses reported that both the nurses and patients became empowered when they were provided opportunities to use their competences. The nurses’ display of trust in their patients’ abilities fostered a positive relationship in which the patients began to act in a more constructive way, leading to mutual respect and a more balanced relationship. This study’s findings resemble those of previous studies that described how BA increased the patients’ participation in their own care (Strand & von Hausswolff-Juhlin, 2015). The current study found that the nurses’ perceived effects of BA touched on several principles that characterize person-centred care, as it’s described by Ekman et al. (2011). The nurses reported that the patients’ narratives were used to develop caring relationships and for care planning. The BA contract was perceived as important for shared decision making, and the transparency of the contract led to more equal relationships. The nurses described how BA supported the patient in being an active player in the decision-making process, which is also central to the concept of person-centred care (Ekman et al., 2011). The Health Foundation (De Silva, 2014) defines person-centred care with four principles: (1) giving people dignity, compassion, and respect; (2) offering coordinated care, support, or treatment, (3) providing personalized care, support, or treatment; and (4) supporting people to recognize and develop their own strengths and abilities to enable them to live an independent and fulfiling life. These principles are encompassed in this study’s findings, which describe BA very differently than the ordinary inpatient care of patients with emotional instability and who self-harm. The nurses said that BA forced them to focus on care instead of organizing care around the pharmacological treatment of the patients. The nurses in our study described how BA supported them in providing health promotive nursing and a recovery-focused nursing practice (Bowen, 2013; Mortimer-Jones et al., 2016). In hindsight, it would have been interesting to explore the nurses’ ideas on how to develop BA, such as how to further increase the patients’ decision making.

## Relevance for clinical practice

Previous studies show that increased training and knowledge have a positive effect on nurses’ attitudes towards working with persons who self-harm (Karman et al., 2015; McHale & Felton, 2010; Saunders et al., 2012). The nurses in our study may have increased their knowledge of working with patients who self-harm and have emotional instability based on the training provided prior to the intervention, and thus some of the positive effects may be attributed to increased knowledge rather than the BA experience itself. Nevertheless, our findings suggest that BA may reduce the work-related stress experienced by nurses working with patients with emotional instability and who self-harm in inpatient care. Consistent with other studies, our findings also suggest that BA improves the inpatient care of such patients (Ellegaard et al., 2017a, 2017b).

Person-centred care is often emphasized in healthcare recommendations (Ekman et al., 2011; Price, Djulbegovic, Biswas, & Chatterjee, 2015; WHO, 2013). However, numerous studies have shown that there is often a gap between an organization’s mission and implemented patient care (Sharma, Bamford, & Dodman, 2015). Ekman et al. (2011) noted that while most care providers appear to endorse person-centred care, this approach has yet to be systematically and consistently implemented in actual care. Ekman et al. (2011) cautioned that healthcare personnel tend to focus on disease-centred care before focusing on the patients’ needs. The nurses in our study described how BA helped them to provide care in a way that may be defined as person-centred.

### Strengths and limitations

The distinctive feature, and also strength, of qualitative research is that it can provide detailed descriptions around intersubjective experiences and identify patterns that can serve as a guide for future research. Many of the means to enhance trustworthiness in qualitative research focus on the use of different perspectives (data sources, methods, investigators, or theories) on the same topic (Polit & Beck, 2016). For example, to enhance trustworthiness, four of the authors were independently involved in the analysis process. However, the present study has some limitations in this respect. First, the informants were quite few and were recruited from one single inpatient care unit. Recruiting informants from this single ward may have biased the data with attitudes and competences that is unit specific and does not entirely reflect nurses’ experiences with BA. Also, only day shift nurses participated in this study. The inclusion of additional units and night shift nurses may have provided a richer description. Second, data was collected by semi-structured interviews, more open and narrative interviews may have resulted in richer data. Despite these limitations, we believe that our findings contribute to understanding how to improve mental health nursing. The study was conducted by a research group with experience working with people with BPD in emergency care, inpatient care, and specialized outpatient care contexts. Although the researchers’ experience working with BA ranged from no experience to substantial experience, the variety of pre-understanding of the phenomena under study among the researchers was considered a strength throughout the research process (Polit & Beck, 2016).

## Conclusions

This study’s findings indicate that BA supports nurses in providing inpatient care that is helpful for patients with emotional instability and self-harm. As a structured nursing intervention in psychiatric care, it is innovative and can inspire clinical practice to further develop and implement person-centred care. Future research should consider the patients’ perspective of BA, its effectiveness regarding psychiatric symptoms, and the health economics of BA.

## References

[CIT0001] American Psychiatric Association (2013). *Diagnostic and statistical manual of mental disorders: DSM-5*. Washington, DC: Author.

[CIT0002] Barker P., & Buchanan-Barker P. (2010). The tidal model of mental health recovery and reclamation: Application in acute care settings. *Issues in Mental Health Nursing*, 31(3), 171–13. 20144029 10.3109/01612840903276696

[CIT0003] Barker P., & Buchanan-Barker P. (2018). The tidal model. Retrieved from http://www.tidal-model.com/

[CIT0004] Betan E., Heim A. K., Zittel Conklin C., & Westen D. (2005). Countertransference phenomena and personality pathology in clinical practice: An empirical investigation. *The American Journal of Psychiatry*, 162(5), 890–898. 15863790 10.1176/appi.ajp.162.5.890

[CIT0005] Black D. W., Blum N., Pfohl B., & Hale N. (2004). Suicidal behavior in borderline personality disorder: Prevalence, risk factors, prediction, and prevention. *Journal of Personality Disorders*, 18(3), 226–239. 15237043 10.1521/pedi.18.3.226.35445

[CIT0006] Bodner E., Cohen-Fridel S., Mashiah M., Segal M., Grinshpoon A., Fischel T., & Iancu I. (2015). The attitudes of psychiatric hospital staff toward hospitalization and treatment of patients with borderline personality disorder. *BMC Psychiatry*, 15, 2. 25609479 10.1186/s12888-014-0380-y PMC4307152

[CIT0007] Bolton J. M., Pagura J., Enns M. W., Grant B., & Sareen J. (2010). A population-based longitudinal study of risk factors for suicide attempts in major depressive disorder. *Journal of Psychiatric Research*, 44(13), 817–826. 20122697 10.1016/j.jpsychires.2010.01.003 PMC2888712

[CIT0008] Bowen M. (2013). Borderline personality disorder: Clinicians’ accounts of good practice. *Journal of Psychiatric and Mental Health Nursing*, 20(6), 491–498. 22727023 10.1111/j.1365-2850.2012.01943.x

[CIT0009] Browne E. M. (2013). Redesigning and retrofitting existing facilities for behavioral healthcare. *Journal of Healthcare Protection Management*, 29(2), 46–50. 24020319

[CIT0010] Carroll R., Metcalfe C., & Gunnell D. (2014). Hospital presenting self-harm and risk of fatal and non-fatal repetition: Systematic review and meta-analysis. *PLoS One*, 9(2), e89944. 24587141 10.1371/journal.pone.0089944 PMC3938547

[CIT0011] Cleary M., Siegfried N., & Walter G. (2002). Experience, knowledge and attitudes of mental health staff regarding clients with a borderline personality disorder. *International Journal of Mental Health Nursing*, 11(3), 186–191. 12510596 10.1046/j.1440-0979.2002.00246.x

[CIT0012] Cutcliffe J. R., & Barker P. (2002). Considering the care of the suicidal client and the case for ‘engagement and inspiring hope’ or ‘observations’. *Journal of Psychiatric and Mental Health Nursing*, 9(5), 611–621. 12358715 10.1046/j.1365-2850.2002.00515.x

[CIT0013] Cutcliffe J. R., Santos J. C., Kozel B., Taylor P., & Lees D. (2015). Raiders of the lost art: A review of published evaluations of inpatient mental health care experiences emanating from the United Kingdom, Portugal, Canada, Switzerland, Germany and Australia. *International Journal of Mental Health Nursing*, 24(5), 375–385. 26300551 10.1111/inm.12159

[CIT0014] De Silva D. (2014). *Helping measure person-centred care*. London Retrieved from http://www.health.org.uk/sites/health/files/HelpingMeasurePersonCentredCare.pdf

[CIT0015] Dickens G. L., Lamont E., & Gray S. (2016). Mental health nurses’ attitudes, behaviour, experience and knowledge regarding adults with a diagnosis of borderline personality disorder: Systematic, integrative literature review. *Journal of Clinical Nursing*, 25(13–14), 1848–1875. 27139693 10.1111/jocn.13202

[CIT0016] Ejneborn Looi G. M., Engström A., & Sävenstedt S. (2015). A self-destructive care: Self-reports of people who experienced coercive measures and their suggestions for alternatives. *Issues in Mental Health Nursing*, 36(2), 96–103. 25625709 10.3109/01612840.2014.951134

[CIT0017] Ekman I., Swedberg K., Taft C., Lindseth A., Norberg A., Brink E., … Sunnerhagen K. S. (2011). Person-centered care–Ready for prime time. *European Journal of Cardiovascular Nursing*, 10(4), 248–251. 21764386 10.1016/j.ejcnurse.2011.06.008

[CIT0018] Ellegaard T., Bliksted V., Lomborg K., & Mehlsen M. (2017a). Use of patient-controlled psychiatric hospital admissions: Patients’ perspective. *Nordic Journal of Psychiatry*, 71(5), 370–377. 28326863 10.1080/08039488.2017.1302505

[CIT0019] Ellegaard T., Mehlsen M., Lomborg K., & Bliksted V. (2017b). Use of patient-controlled psychiatric hospital admissions: Mental health professionals’ perspective. *Nordic Journal of Psychiatry*, 71(5), 362–369. 28318346 10.1080/08039488.2017.1301548

[CIT0020] Elo S., & Kyngäs H. (2008). The qualitative content analysis process. *Journal of Advanced Nursing*, 62(1), 107–115. 18352969 10.1111/j.1365-2648.2007.04569.x

[CIT0021] Gysin-Maillart A., Schwab S., Soravia L., Megert M., & Michel K. (2016). A Novel brief therapy for patients who attempt suicide: A 24-months follow-up randomized controlled study of the attempted suicide short intervention program (ASSIP). *PLoS Medicine*, 13(3), e1001968. 26930055 10.1371/journal.pmed.1001968 PMC4773217

[CIT0022] Happell B., & Harrow A. (2010). Nurses’ attitudes to the use of seclusion: A review of the literature. *International Journal of Mental Health Nursing*, 19(3), 162–168. 20550639 10.1111/j.1447-0349.2010.00669.x

[CIT0023] Helleman M., Goossens P. J., Kaasenbrood A., & van Achterberg T. (2014a). Evidence base and components of brief admission as an intervention for patients with borderline personality disorder: A review of the literature. *Perspectives in Psychiatric Care*, 50(1), 65–75. 24387616 10.1111/ppc.12023

[CIT0024] Helleman M., Goossens P. J., Kaasenbrood A., & van Achterberg T. (2014b). Experiences of patients with borderline personality disorder with the brief admission intervention: A phenomenological study. *International Journal of Mental Health Nursing*, 23(5), 442–450. 24890615 10.1111/inm.12074

[CIT0025] Helleman M., Goossens P. J. J., van Achterberg T., & Kaasenbrood A. (2017). Components of brief admission as a crisis intervention for patients with a borderline personality disorder: Results of a Delphi study. *Journal of the American Psychiatric Nurses Association*, 1078390317728330. doi:10.1177/1078390317728330 28850006

[CIT0026] Helleman M., Lundh L. G., Liljedahl S. I., Daukantaite D., & Westling S. (2018). Individuals’ experiences with brief admission during the implementation of the brief admission skane RCT, a qualitative study. *Nordic Journal of Psychiatry*, 72(5), 380–386. 29703119 10.1080/08039488.2018.1467966

[CIT0027] Heskestad S., & Tytlandsvik M. (2008). Patient-guided crisis admissions for severe psychotic conditions. *Tidsskr Nor Laegeforen*, 128(1), 32–35. 18183054

[CIT0028] Holm H., Björkdahl A., & Björkenstam E. (2011). Compulsory care with a question mark. *Lakartidningen*, 108(34), 1544–1545. 22066161

[CIT0029] Jonsson P. D., Nunstedt H., Berglund I. J., Ahlstrom B. H., Hedelin B., Skärsäter I., & Jormfeldt H. (2014). Problematization of perspectives on health promotion and empowerment in mental health nursing–Within the research network “MeHNuRse” and the Horatio conference, 2012. *International Journal of Qualitative Studies on Health and Well-being*, 9, 22945. 24717267 10.3402/qhw.v9.22945 PMC3982111

[CIT0030] Karlin B. E., & Zeiss R. A. (2006). Best practices: Environmental and therapeutic issues in psychiatric hospital design: Toward best practices. *Psychiatric Services*, 57(10), 1376–1378. 17035554 10.1176/ps.2006.57.10.1376

[CIT0031] Karman P., Kool N., Poslawsky I. E., & van Meijel B. (2015). Nurses’ attitudes towards self-harm: A literature review. *Journal of Psychiatric and Mental Health Nursing*, 22(1), 65–75. 25490929 10.1111/jpm.12171

[CIT0032] Langley G. C., & Klopper H. (2005). Trust as a foundation for the therapeutic intervention for patients with borderline personality disorder. *Journal of Psychiatric and Mental Health Nursing*, 12(1), 23–32. 15720494 10.1111/j.1365-2850.2004.00774.x

[CIT0033] Lieb K., Zanarini M. C., Schmahl C., Linehan M. M., & Bohus M. (2004). Borderline personality disorder. *Lancet*, 364(9432), 453–461. 15288745 10.1016/S0140-6736(04)16770-6

[CIT0034] Linehan M. M. (1993). Dialectical behavior therapy for treatment of borderline personality disorder: Implications for the treatment of substance abuse. *NIDA Research Monograph*, 137, 201–216. 8289922

[CIT0035] Links P. S., Shah R., & Eynan R. (2017). Psychotherapy for borderline personality disorder: Progress and remaining challenges. *Current Psychiatry Reports*, 19(3), 16. 28271272 10.1007/s11920-017-0766-x

[CIT0036] MacLean L. M., Meyer M., & Estable A. (2004). Improving accuracy of transcripts in qualitative research. *Qualitative Health Research*, 14(1), 113–123. 14725179 10.1177/1049732303259804

[CIT0037] McHale J., & Felton A. (2010). Self-harm: What’s the problem? A literature review of the factors affecting attitudes towards self-harm. *Journal of Psychiatric and Mental Health Nursing*, 17(8), 732–740. 21050340 10.1111/j.1365-2850.2010.01600.x

[CIT0038] Michel K., Valach L., & Gysin-Maillart A. (2017). A novel therapy for people who attempt suicide and why we need new models of suicide. *International Journal of Environmental Research and Public Health*, 14(3). doi:10.3390/ijerph14030243 PMC5369079 28257071

[CIT0039] Mortimer-Jones S., Morrison P., Munib A., Paolucci F., Neale S., Bostwick A., & Hungerford C. (2016). Recovery and borderline personality disorder: A description of the innovative open borders program. *Issues in Mental Health Nursing*, 37(9), 624–630. 27327362 10.1080/01612840.2016.1191565

[CIT0040] Newton-Howes G., & Mullen R. (2011). Coercion in psychiatric care: Systematic review of correlates and themes. *Psychiatric Services*, 62(5), 465–470. 21532070 10.1176/ps.62.5.pss6205_0465

[CIT0041] Odelis C., & Ramklint M. (2014). **En nationell kartläggnng av förekomsten av självskadande beteende hos patienter inom barn & ungdoms- och vuxenpsykiatri*n*. Retrieved from Nationella Självskadeprojektet och Uppsala Universitet: https://nationellasjalvskadeprojektet.se/wpcontent/uploads/2016/06/Slutrapportprevalensm%C3%A4tning140410.pdf.

[CIT0042] Papadopoulos C., Ross J., Stewart D., Dack C., James K., & Bowers L. (2012). The antecedents of violence and aggression within psychiatric in-patient settings. *Acta Psychiatrica Scandinavica*, 125(6), 425–439. 22268678 10.1111/j.1600-0447.2012.01827.x

[CIT0043] Paris J. (2002). Chronic suicidality among patients with borderline personality disorder. *Psychiatric Services*, 53(6), 738–742. 12045312 10.1176/appi.ps.53.6.738

[CIT0044] Perseius K. I., Andersson E., Åsberg M., & Samuelsson M. (2006). Health-related quality of life in women patients with borderline personality disorder. *Scandinavian Journal of Caring Sciences*, 20(3), 302–307. 16922984 10.1111/j.1471-6712.2006.00408.x

[CIT0045] Perseius K. I., Ekdahl S., Åsberg M., & Samuelsson M. (2005). To tame a volcano: Patients with borderline personality disorder and their perceptions of suffering. *Archives of Psychiatric Nursing*, 19(4), 160–168. 16088854 10.1016/j.apnu.2005.05.001

[CIT0046] Perseius K. I., Kåver A., Ekdahl S., Åsberg M., & Samuelsson M. (2007). Stress and burnout in psychiatric professionals when starting to use dialectical behavioural therapy in the work with young self-harming women showing borderline personality symptoms. *Journal of Psychiatric and Mental Health Nursing*, 14(7), 635–643. 17880657 10.1111/j.1365-2850.2007.01146.x

[CIT0047] Perseius K. I., Öjehagen A., Ekdahl S., Åsberg M., & Samuelsson M. (2003). Treatment of suicidal and deliberate self-harming patients with borderline personality disorder using dialectical behavioral therapy: The patients’ and the therapists’ perceptions. *Archives of Psychiatric Nursing*, 17(5), 218–227. 14608551 10.1016/s0883-9417(03)00093-1

[CIT0048] Polit D. F., & Beck C. T. (2016). *Nursing research: Generating and assessing evidence for nursing practice* (10th ed.). Philadelphia: Wolters Kluwer.

[CIT0049] Price A. I., Djulbegovic B., Biswas R., & Chatterjee P. (2015). Evidence-based medicine meets person-centred care: A collaborative perspective on the relationship. *Journal of Evaluation in Clinical Practice*, 21(6), 1047–1051. 26358758 10.1111/jep.12434

[CIT0050] Rise M. B., Evensen G. H., Moljord I. E., Ro M., Bjorgen D., & Eriksen L. (2014). How do patients with severe mental diagnosis cope in everyday life - a qualitative study comparing patients’ experiences of self-referral inpatient treatment with treatment as usual? *BMC Health Services Research*, 14, 347. 25127539 10.1186/1472-6963-14-347 PMC4138383

[CIT0051] Saunders K. E., Hawton K., Fortune S., & Farrell S. (2012). Attitudes and knowledge of clinical staff regarding people who self-harm: A systematic review. *Journal of Affective Disorders*, 139(3), 205–216. 21925740 10.1016/j.jad.2011.08.024

[CIT0052] Sharma T., Bamford M., & Dodman D. (2015). Person-centred care: An overview of reviews. *Contemporary Nurse*, 51(2–3), 107–120. 26866531 10.1080/10376178.2016.1150192

[CIT0053] Strand M., Bulik C. M., von Hausswolff-Juhlin Y., & Gustafsson S. A. (2017). Self-admission to inpatient treatment for patients with anorexia nervosa: The patient’s perspective. *International Journal of Eating Disorders*, 50(4), 398–405. 28106920 10.1002/eat.22659

[CIT0054] Strand M., & von Hausswolff-Juhlin Y. (2015). Patient-controlled hospital admission in psychiatry: A systematic review. *Nordic Journal of Psychiatry*, 69(8), 574–586. 25832757 10.3109/08039488.2015.1025835

[CIT0055] Victor S. E., & Klonsky E. D. (2014). Correlates of suicide attempts among self-injurers: A meta-analysis. *Clinical Psychology Review*, 34(4), 282–297. 24742496 10.1016/j.cpr.2014.03.005

[CIT0056] Westwood L., & Baker J. (2010). Attitudes and perceptions of mental health nurses towards borderline personality disorder clients in acute mental health settings: A review of the literature. *Journal of Psychiatric and Mental Health Nursing*, 17(7), 657–662. 20712690 10.1111/j.1365-2850.2010.01579.x

[CIT0057] WHO (2013). *Exploring patient participation in reducing health-care-related safety risks*. Copenhagen, Denmark: World Health Organization.

[CIT0058] World Medical Association (2013). World Medical Association Declaration of Helsinki: Ethical principles for medical research involving human subjects. *JAMA*, 310(20), 2191–2194. 24141714 10.1001/jama.2013.281053

[CIT0059] Zetterqvist M., Lundh L. G., Dahlström O., & Svedin C. G. (2013). Prevalence and function of non-suicidal self-injury (NSSI) in a community sample of adolescents, using suggested DSM-5 criteria for a potential NSSI disorder. *Journal of Abnormal Child Psychology*, 41(5), 759–773. 23344701 10.1007/s10802-013-9712-5

